# Robot-assisted biopsy a bibliometric analysis of global trends, hotspots, and emerging frontiers (2000–2025)

**DOI:** 10.1007/s11701-026-03889-2

**Published:** 2026-08-28

**Authors:** Yanwen Liu, Shanshan Li, Chenggang Liu

**Affiliations:** 1 Jiangning District, Nanjing University of Aeronautics and Astronautics, Jiangjun Road No.29, Nanjing, 211106 China; 2 School of Basic Medical Sciences, Heilongjiang University of Chinese Medicine, 24 Heping Road, Harbin, 150040 China

**Keywords:** Robotic-assisted biopsy, Bibliometric analysis, Prostate cancer, Robotic bronchoscopy, Artificial intelligence

## Abstract

Robot-assisted biopsy techniques achieve precise minimally invasive sampling of deep-seated and anatomically complex lesions through stable robotic manipulation, intelligent path planning, and multimodal imaging fusion. These techniques have accumulated substantial evidence in urology, neurosurgery, and thoracic intervention. However, the field spans multiple organs and technical directions, and lacks a systematic, macroscopic depiction of its overall knowledge structure, evolutionary trajectory, and emerging frontiers**. **This study aims to systematically map global research trends, the distribution of core research actors, the evolution of the knowledge structure, and emerging frontier directions in robot-assisted biopsy via bibliometric methods, thereby providing a macro-level evidence map for researchers, clinicians, and policymakers**. **Using the Web of Science Core Collection as the primary data source, we retrieved literature related to robot-assisted biopsy from 2000 to 2025, limited to English Articles and Reviews; an independent dataset was constructed from PubMed for semantic validation at the MeSH term level. Descriptive statistics, collaboration networks, co-citation networks, keyword co-occurrence, citation burst detection, and dual-map overlay analysis of journals were conducted using Bibliometrix, VOSviewer 1.6.20, CiteSpace 6.4.R1, and Scimago Graphica**. **A total of 2,336 publications were included, with an average annual growth rate of 16.27%. Research progressed through three phases: a slow start from 2000–2009, steady growth from 2010–2016, and accelerated expansion after 2017; publications since 2017 account for 70.85% of the total. The United States (826 publications, 35.4%) ranked first in both productivity and academic impact; China (380 publications, 16.3%) ranked second in output but had a lower citations-per-paper (14.4). Engineering and clinical medicine journals form a dual-core citation structure, with medical imaging journals positioned as interdisciplinary bridging hubs. Keyword analysis showed biopsy(618 occurrences) and medical robotics(612 occurrences) at the network core; prostate cancer, accuracy, and diagnosis constitute the traditional research mainline, while terms such as diagnostic yield, navigation, and artificial intelligence have become increasingly active in recent years. Citation burst analysis indicates a shift in research focus from early robotic system design (2006–2013) to clinical diagnostic paradigms (post-2017), with persistent citation bursts in recent years for pulmonary robotic bronchoscopy literature. MeSH term analysis of the independent PubMed validation set (1,531 publications) further confirmed main research lines including robotic technology, prostate tumors, image-guided biopsy, and pulmonary robotic intervention**. **Robot-assisted biopsy is transitioning from validation of technical feasibility toward creation of clinical value. Prostate biopsy represents the most mature clinical translation pathway; robot-assisted bronchoscopy is the one of the most rapidly growing emerging directions. AI-driven autonomous navigation and human–machine collaboration are reshaping the technological landscape of the field. Future efforts should emphasize multicenter comparative studies, standardized outcome measures, and health-economic evaluations to facilitate integration of robot-assisted biopsy into routine clinical practice.

**Graphical abstract:**

**Figure:**
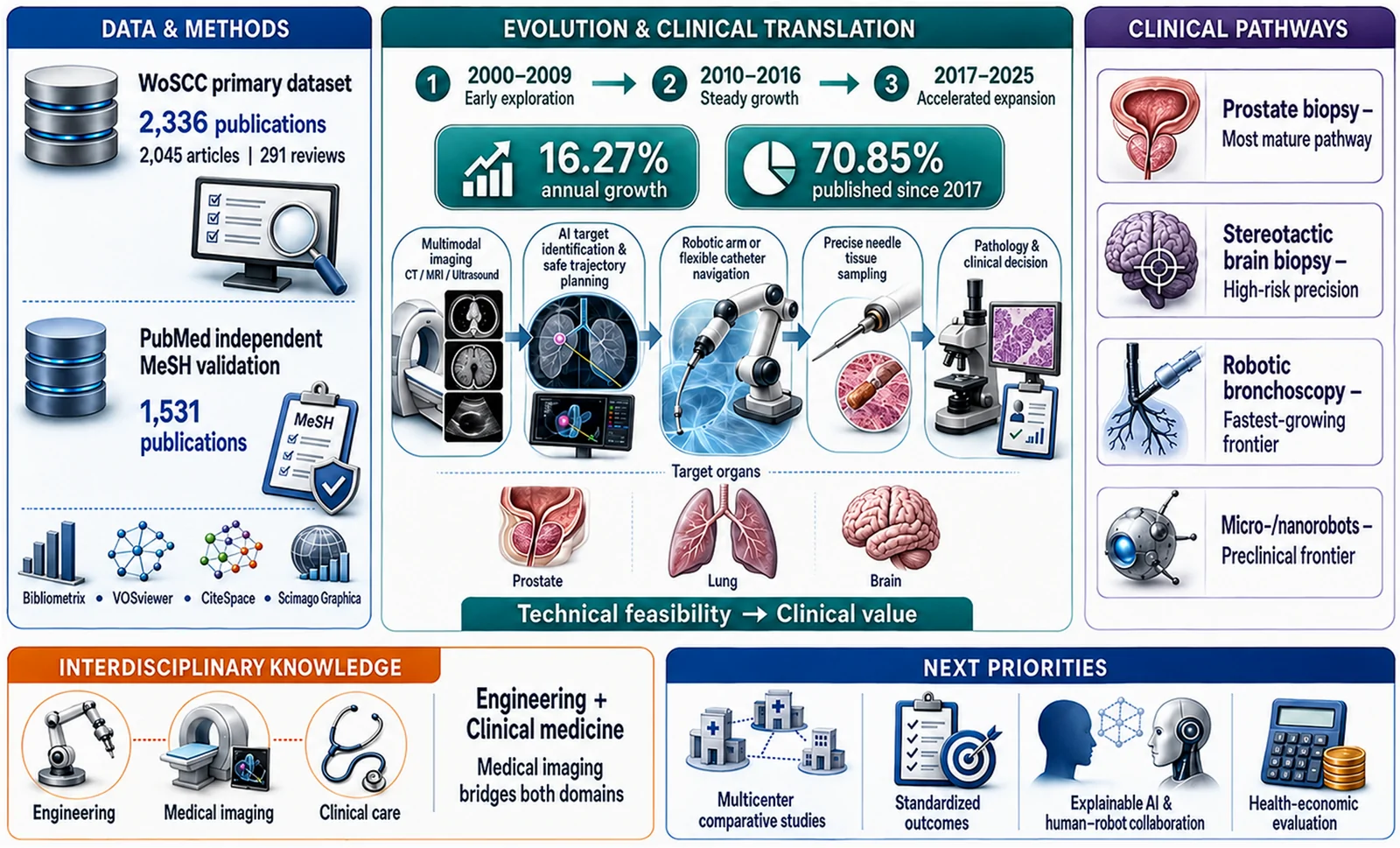

**Supplementary Information:**

The online version contains supplementary material available at https://doi.org/10.1007/s11701-026-03889-2.

## Introduction

Biopsy is the cornerstone for obtaining histopathological diagnoses; its procedural precision and safety directly affect early diagnosis, therapeutic decision-making, and long-term prognosis for oncology patients. However, conventional biopsy methods encounter technical bottlenecks when addressing deep-seated lesions, anatomically complex regions, or lesions adjacent to critical functional areas, often suffering from inaccurate localization, insufficient sampling, and elevated complication risks. The emergence of robot-assisted biopsy technology offers a novel technical pathway to address these clinical challenges. Through the stable manipulation of robotic arms, intelligent trajectory planning, and multi-modal image fusion, it enables precise minimally invasive sampling of lesions that are difficult to reach with traditional methods [1]. Historically, stereotactic brain biopsy was among the earliest application areas for surgical robots.

The first robot-assisted biopsy procedure was performed in 1985, though early systems were largely industrial prototypes retrofit for medical use, costly, and lacking widespread regulatory acceptance [2]. The approval of the da Vinci Surgical System by the U.S. FDA in 2000 marked the formal entry of robotic-assisted surgery into mainstream practice, after which substantial systematic clinical research and academic literature began to emerge [3]. Entering the 2020s, the technology is undergoing profound shifts from purely mechanical assistance toward high degrees of intelligence, autonomy, and miniaturization [4]. Current innovations focus on AI-driven autonomous navigation, multi-modal image fusion, and clinical applications of micro- and nanorobots. In urology, robot-assisted prostate biopsy has become one of the most intensively studied directions [5, 6]; research hotspots have shifted from early feasibility validation to mpMRI-guided fused navigation and AI-assisted decision support. In neurosurgery, robot-assisted stereotactic biopsy has been demonstrated to be an effective and safe method for intracranial lesion sampling.

Meta-analyses further show that robot-assisted brain biopsy has a pooled diagnostic yield of 97%, with diagnostic accuracy and safety comparable to the framed stereotactic biopsy considered the gold standard [7]. In thoracic interventions, robot-assisted bronchoscopy has emerged as a novel minimally invasive technique for diagnosing peripheral pulmonary nodules [8]. Concurrently, the deep integration of artificial intelligence and robotics represents the most striking emerging direction. Studies indicate we are entering an AI-guided era in which AI can automatically analyze images, identify targets and obstacles, compute safe trajectories, and autonomously navigate the biopsy needle to the target with unprecedented precision [9]. The deep embedding of AI is driving robot-assisted biopsy from assisted execution toward autonomous navigation. Although micro- and nanorobot technologies remain primarily at the basic research stage, their potential in targeted drug delivery and minimally invasive diagnostics is substantial and may enable unprecedented levels of minimally invasive intervention.

Despite evidence supporting the efficacy and safety of robot-assisted biopsy across various anatomical sites, a systematic bibliometric mapping of the field’s overall development trajectory, global distribution of research strength, and the evolution of its knowledge structure is still lacking. In recent years, bibliometric analyses have been used to map subfields of robotic surgery; for example, bibliometric studies of robot-assisted prostate biopsy have revealed exponential growth and a shift of research hotspots toward clinically significant prostate cancer detection [6]. Other scholars have conducted knowledge-mapping analyses of robot-assisted stereotactic neurosurgery [10]. However, these studies have been limited to single organs or procedures and have not integrated literature across multiple sites such as the urinary tract, nervous system, thorax, and breast from a global perspective.

Therefore, a macroscopic bibliometric survey of the interdisciplinary area of robot-assisted biopsy holds significant academic value. To this end, the present study performs a systematic search and bibliometric analysis of robot-assisted biopsy literature published in the Web of Science Core Collection and PubMed between 2000 and 2025. We chart the annual publication growth trajectory and the distribution of core countries, institutions, and journals; identify high-frequency keywords and core research themes and their evolution; detect emergence and shifts in research hotspots; and reveal current emerging frontiers and future development directions. The aim is to provide researchers, clinicians, and policymakers in this field with a macroscopic, evidence-based knowledge map and decision-making reference.

## Methodology

### Data sources and search strategy

The Web of Science Core Collection (WoSCC) was used as the primary bibliometric data source. PubMed was analyzed independently to provide supplementary validation based on Medical Subject Headings (MeSH). WoSCC provides complete information on authors, institutions, countries, journals, and cited references, making it suitable for collaboration network, co-citation, and knowledge-mapping analyses. PubMed provides standardized MeSH descriptors and qualifiers, allowing the research themes identified in WoSCC to be validated from a biomedical semantic perspective. The two databases were processed and analyzed separately without cross-database merging or combined bibliometric analysis.

The publication period was restricted to January 1, 2000, through December 31, 2025. The final searches and data exports were completed on May 25, 2026. The search strategy consisted of two concept modules: robotics and biopsy. The Topic field was used in WoSCC, while the PubMed search combined title/abstract terms with MeSH terms. The complete search strategies are provided in Supplementary_Material. The WoSCC search was restricted to English-language Articles and Reviews. The same time, language, and publication-type restrictions were applied in PubMed. Complete PubMed records containing bibliographic information, abstracts, publication types, and MeSH data were exported.

### Literature screening and data processing

**Fig. 1:**
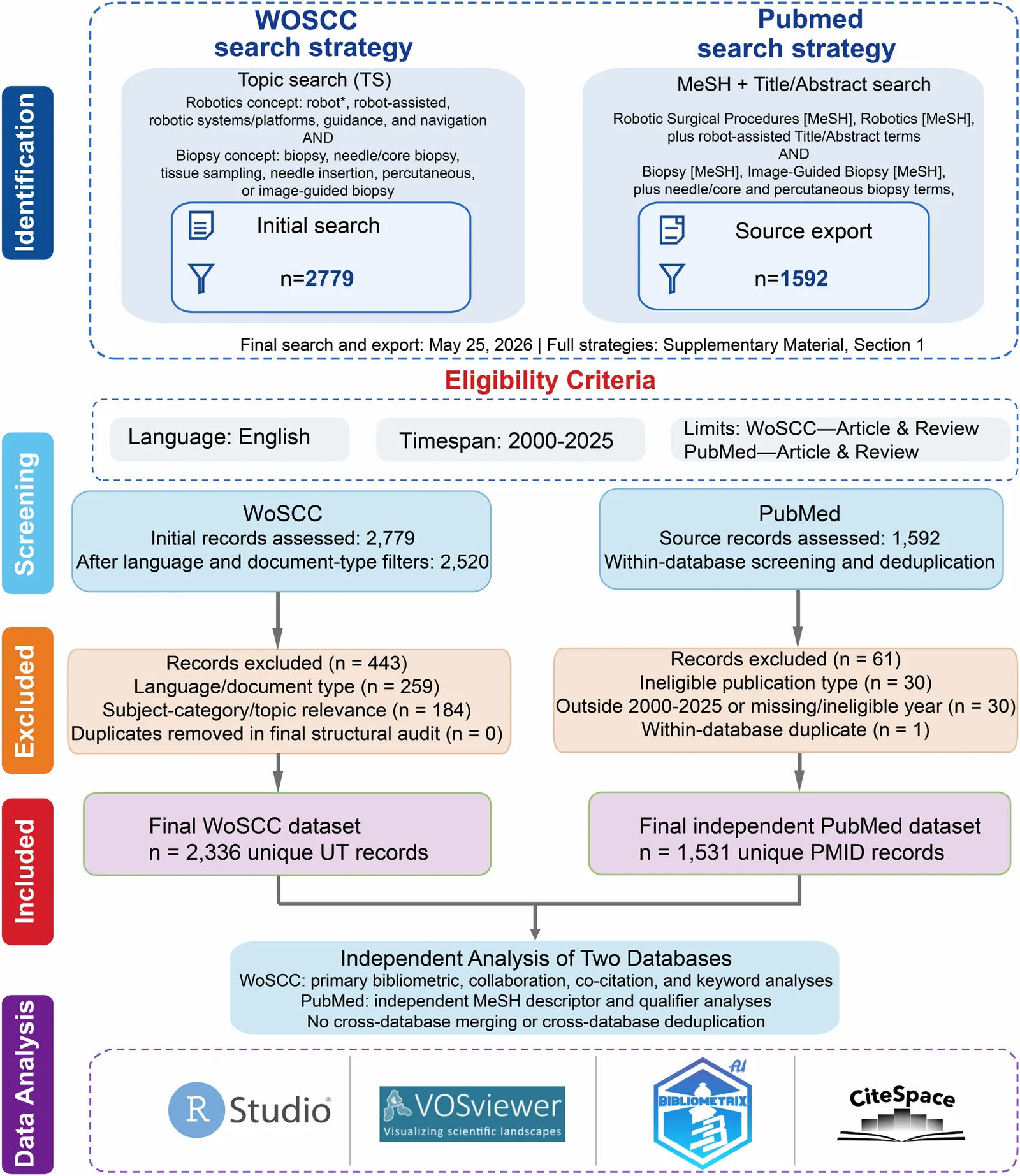
Workflow of the data search strategy

Two investigators independently screened the retrieved records according to predefined eligibility criteria. Studies were included if they directly addressed the design, validation, or clinical application of robotic systems, robotic navigation, or robotic control in tissue biopsy, needle sampling, bronchoscopic sampling, or stereotactic biopsy. Non-research publications, including conference abstracts, editorials, book reviews, news items, letters, and corrections, were excluded. Studies limited to robot-assisted treatment, liquid biopsy, automated laboratory testing, or conventional non-robotic puncture procedures were also excluded. Full texts were reviewed when the titles and abstracts provided insufficient information. Disagreements were resolved through discussion. Detailed inclusion and exclusion criteria are provided in Supplementary_Material, and the literature-screening process is shown in Fig. 1.

WoSCC records were imported into the R(4.5.1) environment and processed using Bibliometrix for format conversion and quality assessment. The author, institution, country, journal, and author-keyword fields were standardized. Variations in author names were checked using institutional affiliations and research areas. Institution names were standardized at the main university, hospital, or research-organization level, while country and journal names were merged under their standard forms. A customized thesaurus was used for keyword cleaning. Differences in capitalization, singular and plural forms, abbreviations, hyphenation, and spelling were standardized, and generic terms with limited thematic value were removed. The cleaned data were independently reviewed by two investigators. The standardization procedures and cleaning results are provided in Supplementary_Material.

### Bibliometric and knowledge-mapping analyses

Bibliometrix^[11]^version 4.3.3 was used to calculate annual publication output, annual growth rate, citation indicators, collaboration indicators, and the scientific output of leading countries, institutions, authors, and journals. The influence of countries, institutions, and authors was evaluated using publication count, total citations, average citations per publication, and the H-index. A three-field plot was used to present the relationships among authors, author keywords, and source journals. Annual and cumulative publication output, average citations per publication, and annual total citations were visualized in R(4.5.1). Quadratic polynomial fitting was used only to describe historical trends and was not used to predict future publication output.

VOSviewer [12] version 1.6.20 was used to construct country-, institution-, and author-level collaboration networks, as well as author, journal, and reference co-citation networks, source-journal citation networks, and author-keyword co-occurrence networks. Full counting and association-strength normalization were applied to all networks. Node size represented publication output, occurrence frequency, or co-citation frequency. Links indicated relationships between nodes, and link thickness reflected the strength of these relationships. Different colors represented network clusters. Total link strength (TLS) was used to measure the overall connection of a node with other nodes in the network. The keyword co-occurrence network was based on cleaned author keywords, with a minimum occurrence threshold of 10. The high-frequency term table and cumulative-frequency analysis were based on the cleaned combined corpus of author keywords and Keywords Plus.

CiteSpace [13] version 6.4.R1 was used to detect reference citation bursts and generate the journal dual-map overlay. The analysis period was set to 2000–2025, with a time slice of one year. Nodes were selected using the g-index with k=25, and the networks were pruned using Pathfinder and pruning sliced networks. Citation-burst analysis was used to identify references that received rapidly increasing attention during specific periods. The journal dual-map overlay was used to show knowledge flows between citing and cited journal disciplines. Scimago Graphica [14] version 1.0.55 was used to visualize the global distribution of publications and international collaboration.

Research hotspots and emerging frontiers were identified by integrating multiple analytical results. VOSviewer was used to identify keyword co-occurrence structures, while Bibliometrix and R were used to calculate the annual and cumulative frequencies of major terms. Thematic evolution was assessed according to the first appearance, median active year, and annual distribution of each term. Two investigators interpreted and named the keyword clusters and research frontiers based on representative publications, citation bursts, and co-citation structures. Detailed thresholds, clustering settings, and visualization parameters are provided in Supplementary_Material.

### Independent pubMed MeSH validation

The PubMed dataset was not merged with the primary WoSCC dataset. Instead, it was analyzed independently as a supplementary biomedical dataset. Bibliometrix was used to describe the general characteristics of the PubMed dataset, and VOSviewer was used to construct MeSH co-occurrence and overlay visualization networks. In the overlay map, node size represented the frequency of a MeSH term, links indicated co-indexing relationships, and node color represented the average publication year of the associated records.

MeSH qualifiers were further extracted and classified into semantic domains based on their biomedical meanings. These domains included biopsy and operative procedures, robotics and enabling technologies, imaging and navigation, neoplasms and anatomical targets, tumor staging and pathology, and outcomes and safety. A heatmap was used to show the distribution of MeSH qualifiers across these semantic domains, while a lollipop chart was used to present the most frequent MeSH terms. The PubMed analysis was used to validate the major research themes identified in WoSCC, including robotics, prostate diagnosis and treatment, image-guided biopsy, and robot-assisted pulmonary interventions. Detailed procedures for MeSH cleaning, qualifier extraction, and semantic-domain classification are provided in Supplementary_Material.

## Results

### Global publication trends and overall characteristics

**Fig. 2:**
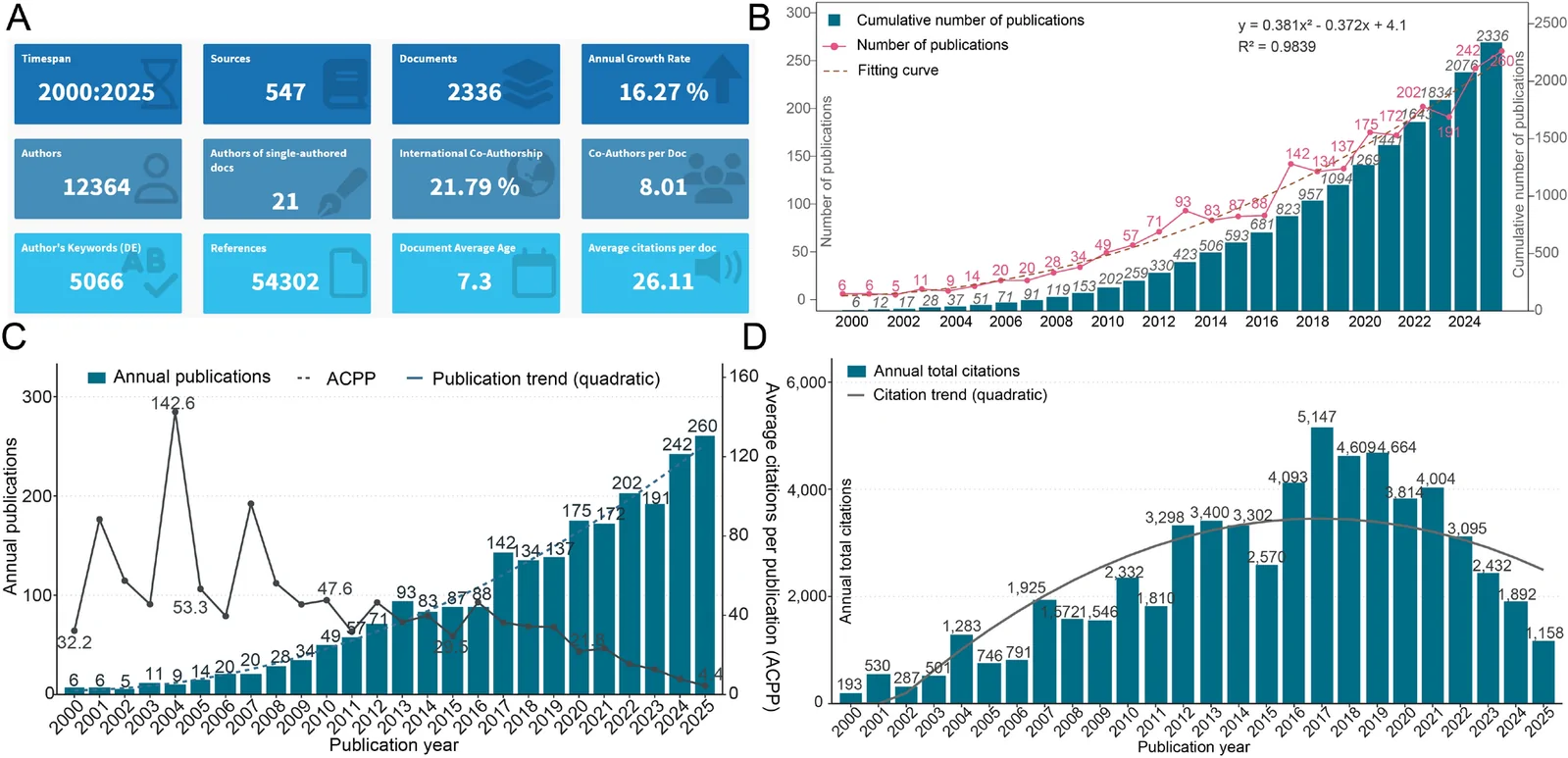
(**A**) Overall characteristics of included publications; (**B**) annual publications and cumulative publication trends; (**C**) annual publications and average citations per article; (**D**) annual total citations and its fitted trend

This study included 2,336 publications related to robot-assisted biopsy, of which 2,045 were Articles and 291 were Reviews. The compound annual growth rate in this field was 16.27%; the average number of authors per article was 8.01; and international collaborative papers accounted for 21.79% (Fig. 2A). Annual publication output showed an overall accelerating upward trend and can be roughly divided into three phases. From 2000 to 2009 the field was in an early exploratory stage, with annual publications rising slowly from 6 to 34. From 2010 to 2016 it entered a steady growth stage, with annual publications increasing from 49 to 88 and reaching a phase peak of 93 in 2013. After 2017, related research accelerated markedly, with annual publications rising from 142 to 202 in 2022. Although there was a brief decline to 191 publications in 2023, 2024 and 2025 saw new highs of 242 and 260 publications, respectively. Publications from 2017–2025 accounted for 70.85% of all publications (Fig. 2B). Average citations per article fluctuated considerably in the early period of the field, reaching a maximum of 142.56 in 2004, and thereafter showed a general downward trend, reaching 4.45 in 2025(Fig. 2C). Cumulative annual citations of papers by publication year rose overall to 5,147 in 2017, remained at a relatively high level thereafter, and have declined gradually in recent years (Fig. 2D).

### Distribution of countries and institutions and collaboration networks

**Fig. 3:**
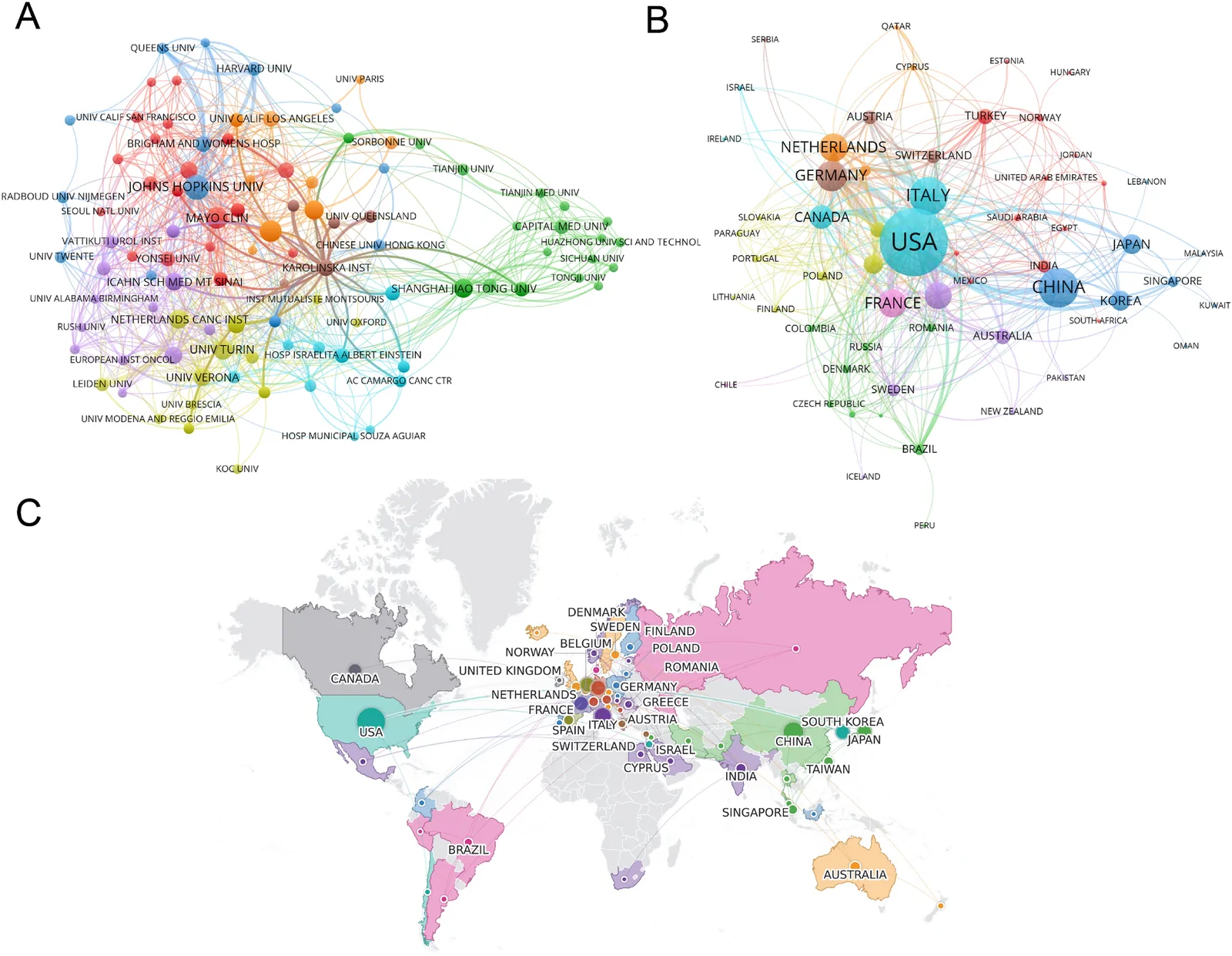
(**A**) Institutional collaboration network.(**B**) Country/region collaboration network.(**C**) Global distribution of publications and international collaboration

Figure 3A shows the collaboration network among major research institutions. Johns Hopkins University lies at the core of the network, with the highest total link strength (TLS = 107), followed by the Mayo Clinic (TLS = 60) and the University of Southern California (TLS = 58). North American and European institutions constitute the main body of the collaboration network. Chinese institutions such as Shanghai Jiao Tong University, Capital Medical University, Tianjin University, and Tianjin Medical University form a relatively concentrated collaborative cluster and connect with research institutions in other countries through key nodes like Shanghai Jiao Tong University. In terms of publication volume, Johns Hopkins University ranks first with 53 publications, followed by Tianjin University and Yonsei University with 37 and 30 publications, respectively. Johns Hopkins University also leads in total citations, citations per paper, and H-index, with 4,238 citations, 80 citations per paper, and an H-index of 34. The top-ranked institution accounts for only 2.3% of all publications, indicating that institutional output in this field is generally dispersed (Table 1).

**Table 1:** Top 10 most productive institutions

Rank	Institution (Country)	Publications (%)	Citations	Avg. citations	H-index
1	Johns Hopkins University (USA)	53 (2.3%)	4,238	80	34
2	Tianjin University (China)	37 (1.6%)	616	16.6	14
3	Yonsei University (South Korea)	30 (1.3%)	489	16.3	15
4	Imperial College London (United Kingdom)	27 (1.2%)	859	31.8	18
5	Mayo Clinic (USA)	25 (1.1%)	750	30	13
6	Shanghai Jiao Tong University (China)	24 (1.0%)	386	16.1	11
7	Harbin University of Science & Technology (China)	24 (1.0%)	157	6.5	8
8	Chinese University of Hong Kong (China)	21 (0.9%)	360	17.1	8
9	University of Verona (Italy)	18 (0.8%)	284	15.8	10
10	Memorial Sloan Kettering Cancer Center (USA)	17 (0.7%)	768	45.2	14

Figure 3B shows that the United States is at the center of the country-level collaboration network, collaborating with 48 countries and having the highest total link strength (TLS = 466), followed by Italy (TLS = 285), Germany (TLS = 188), France (TLS = 162), and the Netherlands (TLS = 157). The United States maintains close collaborative ties with Italy, Germany, the Netherlands, France, Canada, and China; European countries also display dense regional collaborations among themselves. China, together with Japan, South Korea, and other East Asian countries, forms a relatively concentrated collaborative cluster and links to the global collaboration network via the United States and some European countries. Although China ranks second in publication volume, its international collaboration strength remains lower than that of the United States and some European countries. Figure 3C indicates that research on robot-assisted biopsy is primarily concentrated in North America, Western Europe, and East Asia. The United States has the highest number of publications, with 826 papers, accounting for 35.4% of all publications; China ranks second with 380 papers, followed by Italy, Germany, the Netherlands, and France (Table 2). The United States also has the highest total citations and H-index, with 29,851 citations and an H-index of 84.

**Table 2:** Top 10 most productive countries/regions

Rank	Country	Publications (%)	Citations	Avg. citations	H-index
1	USA	826 (35.4%)	29,851	36.1	84
2	China	380 (16.3%)	5,465	14.4	35
3	Italy	226 (9.7%)	6,512	28.8	41
4	Germany	161 (6.9%)	4,780	29.7	35
5	Netherlands	151 (6.5%)	4,690	31.1	40
6	France	147 (6.3%)	3,758	25.6	34
7	United Kingdom	131 (5.6%)	2,992	22.8	33
8	Japan	131 (5.6%)	1,836	14	22
9	South Korea	129 (5.5%)	2,269	17.6	25
10	Canada	124 (5.3%)	4,895	39.5	38

### Core author analysis

**Fig. 4:**
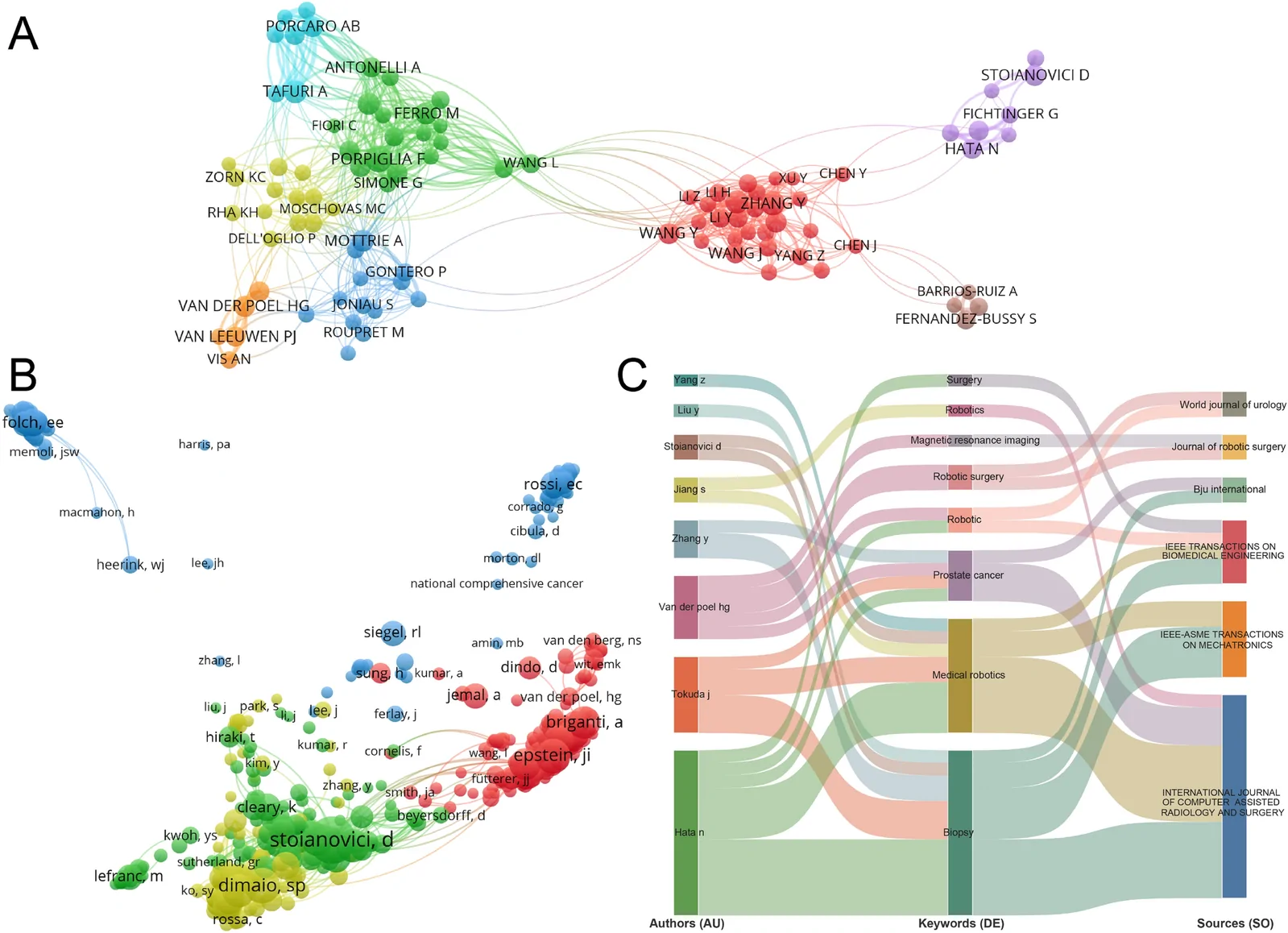
**A**) Author collaboration network.(**B**) Co-cited author network.(**C**) Three-field plot of authors, author keywords, and source journals

Figure 4A displays the collaboration network among major authors. Porpiglia F has the highest total link strength, with Tafuri A and Veccia A tied for second, followed by Antonelli A and Porcaro AB. A European author cluster centered on Porpiglia F, Ferro M, and Antonelli A shows relatively close internal collaboration; a separate major cluster from China—represented by Zhang Y, Wang Y, Li Y, and Yang Z—also forms; Stoianovici D, Hata N, and Fichtinger G comprise the team focused on robotics and image-guided research. While there are connections between clusters, collaboration remains primarily regional and within teams. In terms of publication counts, Jiang S ranks first with 24 papers, followed by Hata N, Tokuda J, Yang Z, and Stoianovici D. Hata N has the highest H-index at 18, while Tokuda J and Stoianovici D each have an H-index of 16 (Table 3).

**Table 3:** Top 10 most productive authors

Author	Articles	Country	H-index	Affiliation	Publication year started
JIANG S	24	CHINA	11	Tianjin University	2012
HATA N	22	USA	18	Brigham & Women’s Hospital	2008
TOKUDA J	21	USA	16	Brigham & Women’s Hospital	2008
YANG Z	20	CHINA	10	Tianjin University	2014
STOIANOVICI D	19	USA	16	Johns Hopkins University	2006
IORDACHITA I	18	USA	13	Johns Hopkins University	2008
ZHANG Y	18	CHINA	6	Harbin University of Science & Technology	2015
PORPIGLIA F	17	ITALY	11	University of Turin	2016
VAN DER POEL HG	16	NETHERLANDS	11	Netherlands Cancer Institute	2012
VAN LEEUWEN FWB	16	NETHERLANDS	11	Leiden University	2012

Figure 4B reveals four major clusters. Stoianovici D is the most influential co-cited author in the network, with 352 co-citations and the highest total link strength (TLS = 6,972). DiMaio SP and Epstein JI follow with 199 and 188 co-citations, respectively; Abolhassani N and Krieger A have 170 and 167 co-citations, respectively. An author group centered on Stoianovici D, DiMaio SP, Krieger A, and Fichtinger G constitutes a key knowledge base for robotic systems and image-guidance technologies; Epstein JI, Briganti A, and D’Amico AV primarily form the clinical research cluster on prostate cancer; Folch EE, Memoli JSW, and Heerink WJ form a knowledge cluster related to lung biopsy and respiratory interventions.

Figure 4C shows the associations among core authors, author keywords, and source journals. Hata N has the broadest topical connections, mainly linked to biopsy and medical robotics; Tokuda J is likewise closely associated with these two topics. Van der Poel HG mainly connects clinical topics such as prostate cancer, robotic surgery, and magnetic resonance imaging. Overall, biopsy is the keyword with the strongest association, followed by medical robotics and prostate cancer. Related research is primarily published in journals in the fields of medical robotics, mechatronics, and biomedical engineering, and also appears in urology journals such as BJU International, Journal of Robotic Surgery, and World Journal of Urology, reflecting the parallel development of engineering technologies and clinical applications in this field.

### Core journals and co-citation analysis

**Fig. 5:**
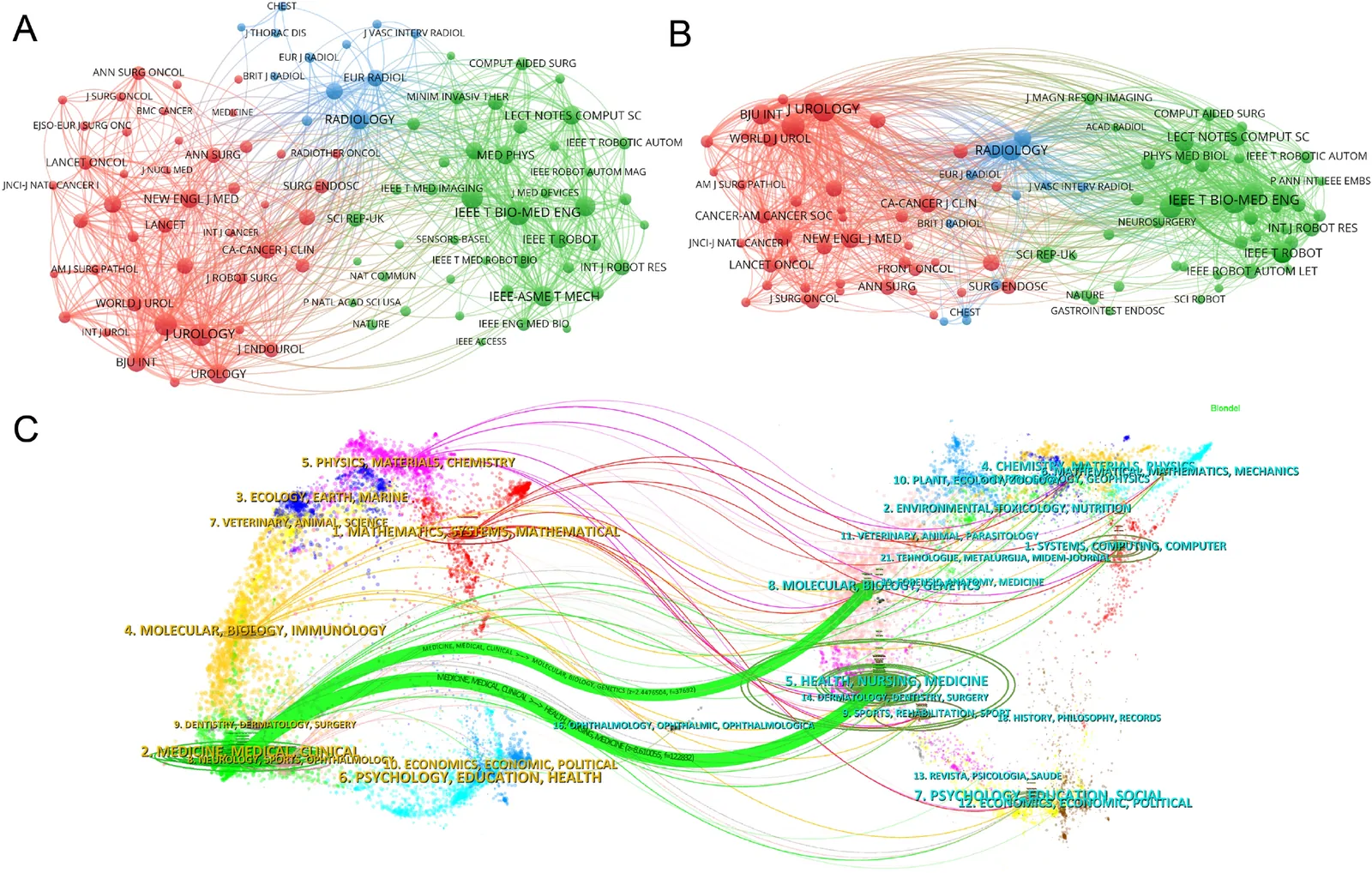
(**A**) Citation network of publication sources.(**B**) Co-citation network of cited sources.(**C**) Dual-map overlay of citing and cited journals

Figure 5A shows three major coalescent clusters: clinical urology and oncology; robotics and biomedical engineering; and medical imaging and interventional radiology. The clinical cluster is centered on Journal of Urology, European Urology, BJU International, and Urology; the engineering and technology cluster primarily includes IEEE Transactions on Biomedical Engineering, IEEE/ASME Transactions on Mechatronics, IEEE Transactions on Robotics, and International Journal of Medical Robotics and Computer Assisted Surgery; the imaging cluster is represented by Radiology, European Radiology, and Journal of Vascular and Interventional Radiology. Journal of Urology has the highest total link strength, followed by European Urology and IEEE Transactions on Biomedical Engineering, indicating that urology and engineering journals jointly occupy the core of the citation network in this field. In terms of publication count, International Journal of Medical Robotics and Computer Assisted Surgery ranks first with 80 articles, followed by Asian Journal of Surgery (52 articles), while BJU International and International Journal of Computer Assisted Radiology and Surgery each published 50 articles (Table 4).

**Table 4:** Top 10 productive and co-cited journals

Rank	Journal	Publications (%)	JIF 2025	Q	Co-cited journal	Co-citation
1	International Journal of Medical Robotics and Computer Assisted Surgery	80 (3.42%)	2.4	Q2	European Urology	3568
2	Asian Journal of Surgery	52 (2.23%)	5.2	Q1	Journal of Urology	3392
3	BJU International	50 (2.14%)	4.1	Q1	BJU International	1635
4	International Journal of Computer Assisted Radiology and Surgery	50 (2.14%)	2.8	Q1	Urology	1487
5	Journal of Endourology	46 (1.97%)	3.2	Q2	Gynecologic Oncology	1307
6	IEEE/ASME Transactions on Mechatronics	45 (1.93%)	6.3	Q1	IEEE Transactions on Biomedical Engineering	1171
7	World Journal of Urology	42 (1.80%)	3.3	Q1	Radiology	1093
8	Journal of Robotic Surgery	41 (1.76%)	4.2	Q1	IEEE/ASME Transactions on Mechatronics	1053
9	IEEE Robotics and Automation Letters	40 (1.71%)	5.3	Q2	Journal of Clinical Oncology	868
10	IEEE Transactions on Biomedical Engineering	40 (1.71%)	4.4	Q2	International Journal of Medical Robotics and Computer Assisted Surgery	857

Figure 5B reveals three interrelated disciplinary groups. European Urology has the highest co-citation frequency at 3,568, followed by Journal of Urology, BJU International, and Urology. Among engineering journals, IEEE Transactions on Biomedical Engineering and IEEE/ASME Transactions on Mechatronics were co-cited 1,171 and 1,053 times, respectively; Radiology was co-cited 1,093 times and occupies a central position among imaging journals.

Figure 5Cfurther illustrates the interdisciplinary citation pathways in the field. Citing journals are mainly distributed across the Medicine, Medical, Clinical and Dentistry, Dermatology, Surgery clusters, and also involve engineering and basic science areas such as Physics, Materials, Chemistry and Mathematics, Systems, Mathematical. Cited journals are mainly concentrated in Health, Nursing, Medicine, Molecular, Biology, Genetics, and Systems, Computing, Computer clusters. The principal citation paths flow from clinical medicine and surgery toward health medicine, molecular biology and genetics, and computing and systems sciences, indicating that research on robot-assisted biopsy is founded on a deep integration of clinical needs, medical imaging, robotic control, and biomedical engineering.

**Fig. 6:**
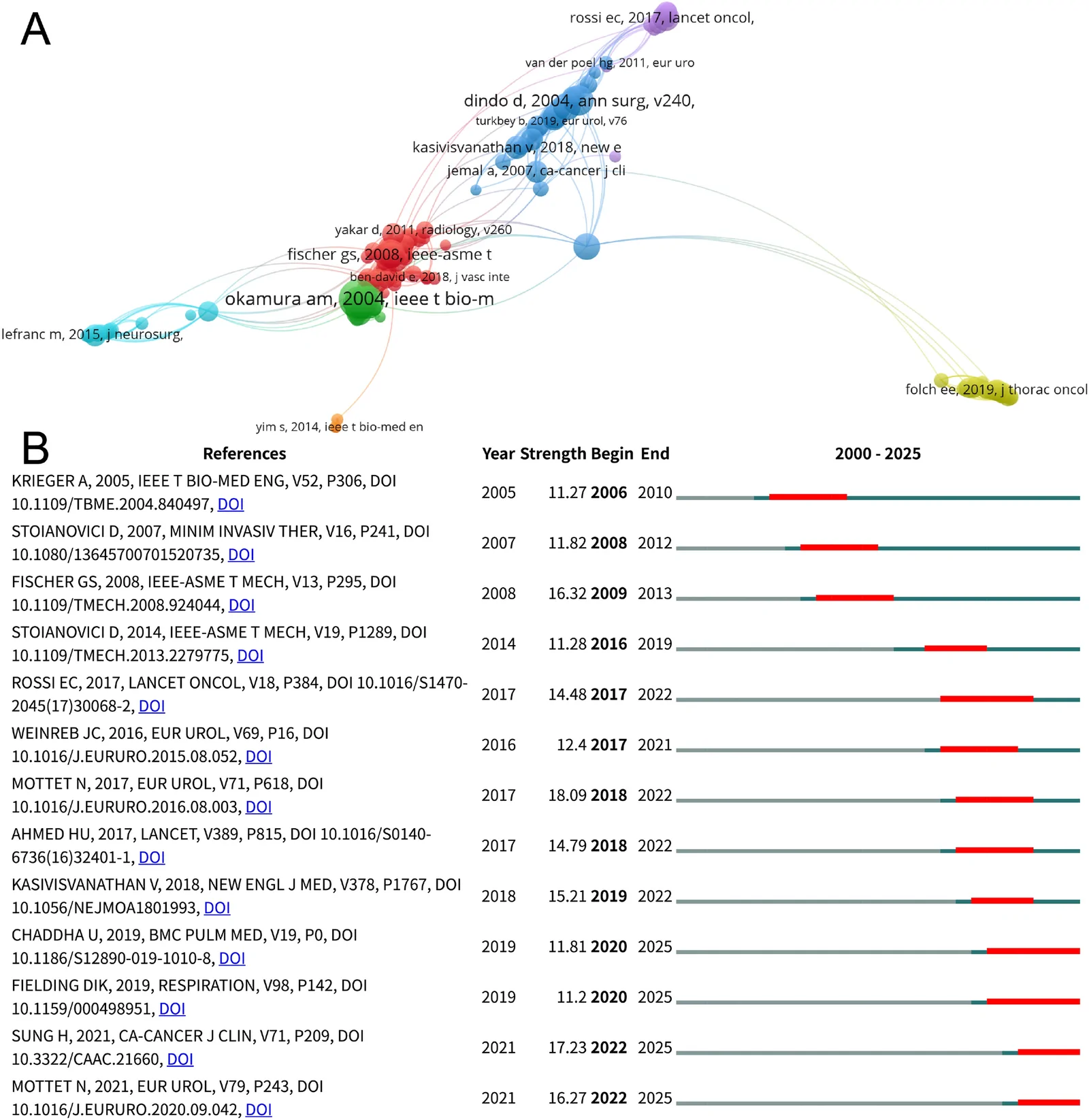
(**A**) Co-citation network of cited references.(**B**) Top 15 references with the strongest citation bursts

Figure 6A shows that the co-cited literature clusters mainly around robotic systems and image guidance, needle–tissue interaction, targeted prostate cancer biopsy, and pulmonary interventions, with engineering works by Okamura AM and others forming an important knowledge base in this field. Figure 6B shows that early burst literature primarily focused on robotic system design and MRI-guided interventions, shifting after 2017 toward prostate cancer imaging diagnosis, targeted biopsy, and clinical guidelines; more recent sustained burst literature mainly involves pulmonary nodule navigation and robot-assisted bronchoscopy, reflecting a shift in research emphasis from technology development to clinical diagnostic application.

## Research hotspots and thematic evolution

**Fig. 7:**
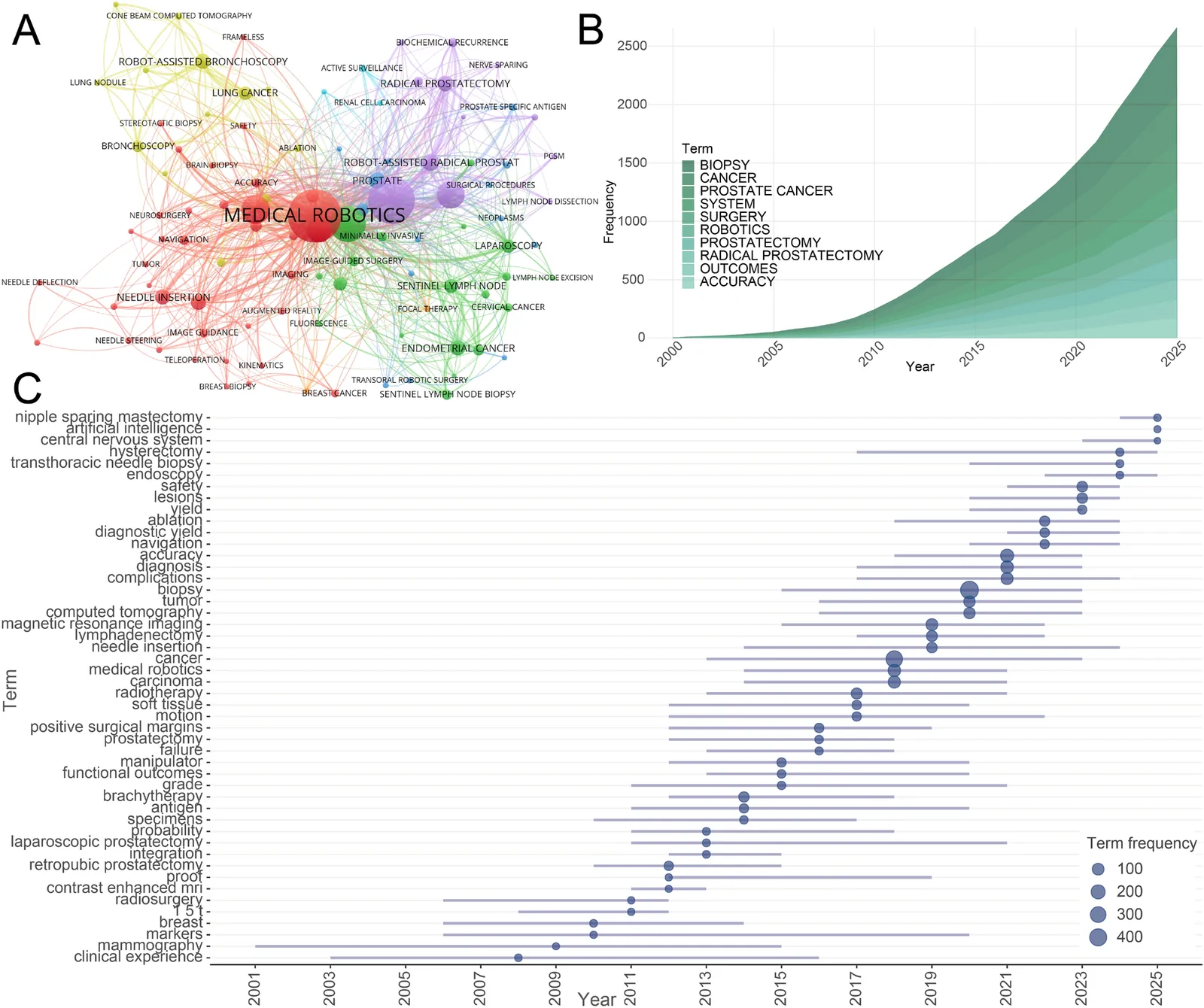
(**A**) Keyword co-occurrence network.(**B**) Cumulative frequency trends of the top 10 keywords.(**C**) Temporal evolution of research topics

Figure 7A shows that 100 high-frequency keywords formed seven clusters, mainly involving robot-assisted puncture and image navigation, robot-assisted prostate cancer surgery, robotic bronchoscopy, lymph node biopsy, and other tumor diagnosis and treatment. Medical robotics and prostate cancer are located at the network center. Table 5 shows that biopsy and medical robotics have the highest occurrence frequencies, at 618 and 612 occurrences respectively, followed by "prostate cancer" (443), cancer (396), and robot-assisted surgery (294). Figure 7B indicates that the cumulative frequency of high-frequency keywords increased markedly after 2010, with biopsy, cancer and prostate cancer continuing to dominate. Research focus has shifted from early breast imaging, radiosurgery, and prostate surgery toward medical robotics, magnetic resonance imaging, puncture accuracy, and image-guided biopsy; in recent years, diagnostic yield, navigation, safety, endoscopy, transthoracic needle biopsy, and artificial intelligence have gradually become active, indicating that robotic bronchoscopy, intelligent navigation, and precise pulmonary biopsy are emerging research frontiers (Fig. 7C).

**Table 5:** Top 20 most frequently occurring keywords

Rank	Keyword	Occurrences	Rank	Keyword	Occurrences
1	biopsy	618	11	carcinoma	126
2	medical robotics	612	12	complications	123
3	prostate cancer	443	13	needle insertion	123
4	cancer	396	14	ultrasound	107
5	robot-assisted surgery	294	15	computed tomography	106
6	magnetic resonance imaging	223	16	tumor	100
7	prostatectomy	190	17	robot-assisted radical prostatectomy	97
8	accuracy	172	18	lymphadenectomy	90
9	radical prostatectomy	169	19	prostate	90
10	diagnosis	150	20	radiotherapy	88

### PubMed MeSH semantic analysis

**Fig. 8:**
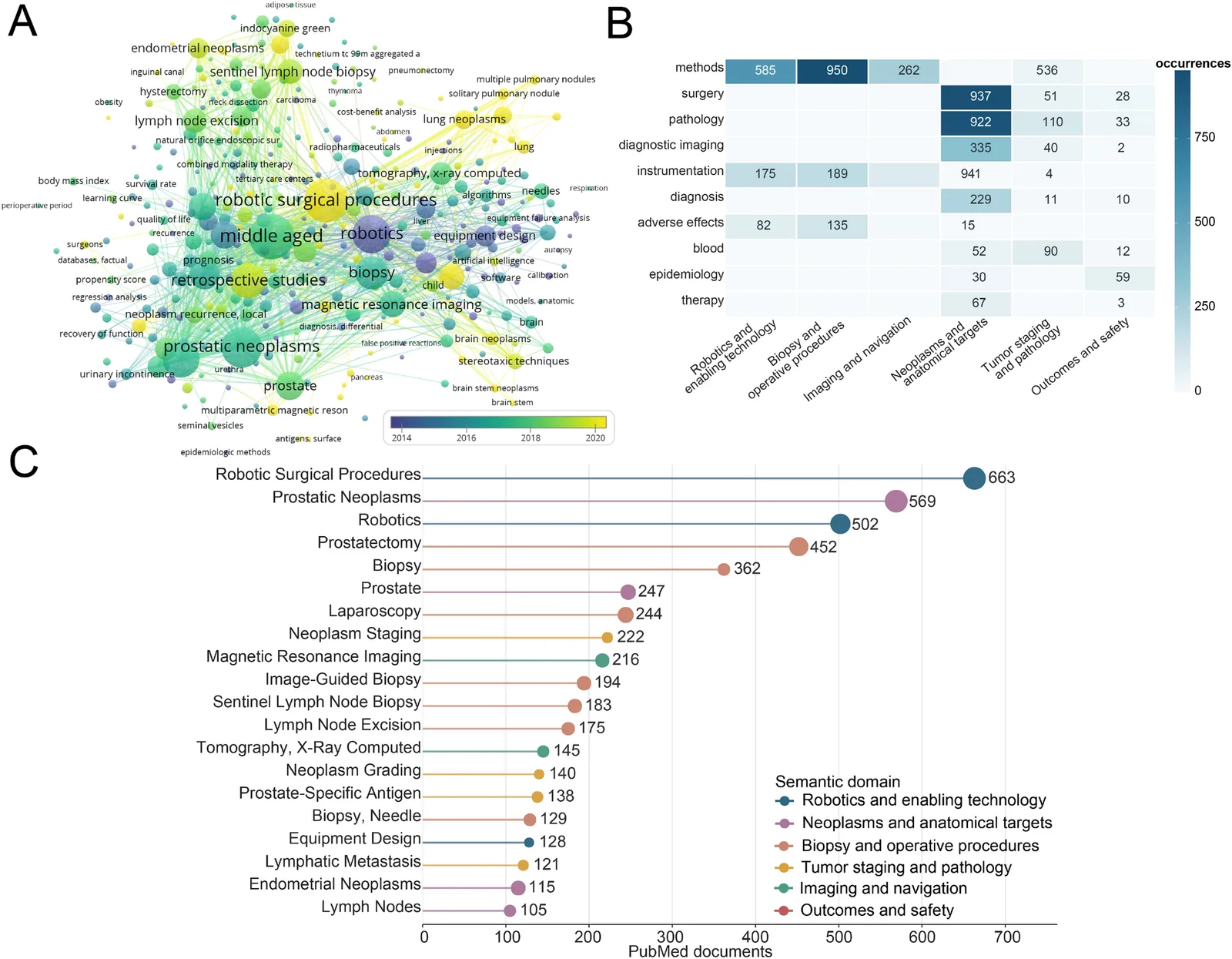
**A**) MeSH co-occurrence network; (**B**) MeSH qualifier heatmap; (**C**) MeSH terms

The independent PubMed dataset included 1,531 publications, comprising 1,346 research articles and 185 reviews (Table 6). Figure 8A shows that MeSH terms form four interrelated clusters, primarily centered on robotic technology, prostate tumors, image-guided biopsy, and other tumor surgeries. In recent years, topics such as bronchoscopy, lung neoplasms, pulmonary nodules, and cone-beam computed tomography have become increasingly active, indicating a research shift toward robot-assisted pulmonary biopsy. Figure 8B indicates that methods are mainly distributed across biopsy and surgical procedures, robotic technology, and image navigation, while surgery and pathology are concentrated on tumors and anatomical targets. Figure 8C shows that Robotic Surgical Procedures has the highest frequency (663 occurrences), followed by Prostatic Neoplasms(569), Robotics(502), Prostatectomy(452), and Biopsy(362). Additionally, Image-Guided Biopsy and Sentinel Lymph Node Biopsy occur 194 and 183 times, respectively. These results further confirm that the field is centered on robotic technology, prostate diagnosis and treatment, and image-guided biopsy, with gradual expansion toward precise pulmonary biopsy.

**Table 6:** General characteristics of the PubMed dataset

Description	Results
MAIN INFORMATION ABOUT DATA	
Timespan	2000:2025
Sources (Journals, Books, etc.)	406
Documents	1531
Annual Growth Rate %	13.19
Document Average Age	8.06
AUTHORS	
Authors	7978
Authors of single-authored docs	16
AUTHORS COLLABORATION	
Single-authored docs	17
Co-Authors per Doc	7.53
Internationally co-authored documents, n (%)	13.13%
DOCUMENT TYPES	
article	1346
review	185

## Discussion

### Global research trends and stages of technological evolution

This bibliometric analysis revealed a clear structural shift in robot-assisted biopsy research. The focus has gradually moved from feasibility studies dominated by engineering and computer science toward the creation of clinical value driven by medical needs. This shift is more than a simple change in research hotspots. It reflects a broader transformation in how knowledge is produced in this field. Early studies mainly addressed robot kinematics, needle–tissue interaction modeling, image registration, and system integration. This is supported by the high co-citation frequency of engineering journals such as IEEE Transactions on Biomedical Engineering and IEEE/ASME Transactions on Mechatronics. These studies established the technical basis of robot-assisted biopsy and demonstrated that robotic systems could guide biopsy needles to predefined targets with acceptable accuracy.

As the technology matured, attention increasingly shifted toward clinical applications. As a diagnostic intervention, the value of robot-assisted biopsy depends not only on whether the needle can reach its target but also on whether the procedure improves diagnostic accuracy, patient safety, and clinical decision-making [15]. This transition is reflected in the evolution from the earlier emphasis on accuracy toward more recent themes such as diagnostic yield, safety, and complications.

The recent rise of artificial intelligence (AI)-related terms further supports this transition. AI is moving the field from mechanical assistance toward cognitive assistance. Robotic systems are no longer limited to executing predefined trajectories but are beginning to participate in target identification, image interpretation, and path optimization [9]. Robot-assisted biopsy is therefore progressing from procedural automation toward decision-level intelligence. After nearly two decades of technological development, feasibility is no longer the main barrier. The key challenge is now the quality of clinical evidence and whether robotic systems can consistently improve diagnostic outcomes, reduce complications, and support treatment decisions in real-world patient populations [16]. This may explain why recent citation bursts have been driven mainly by clinical guidelines and large prospective trials rather than by technical reports alone.

### Organization and flow of interdisciplinary knowledge

The knowledge structure of robot-assisted biopsy shows a dual-core pattern in which engineering and clinical medicine serve as the two main sources of knowledge, while medical imaging provides a functional bridge between them. This structure is evident in both the journal co-citation and keyword co-occurrence networks. Engineering journals, represented by IEEE/ASME Transactions on Mechatronics and IEEE Transactions on Biomedical Engineering, provide the methodological foundation of the field, including robot kinematics, image registration, force sensing, and path-planning algorithms. These technologies can be transferred across organs and clinical settings. Once validated, a needle–tissue interaction model or image-fusion algorithm may be applied to the prostate, lung, brain, and other anatomical sites. This transferability places engineering research upstream in the knowledge-production process [17]. Clinical journals, represented by European Urology and Journal of Urology, define clinical problems, establish outcome measures, and evaluate the practical value of new technologies [18]. Questions such as whether a system improves the detection of clinically significant prostate cancer or reduces the risk of pneumothorax provide clear targets for engineering development [19, 20].

The journal dual-map overlay showed an asymmetric pattern of knowledge flow, with clinical publications drawing extensively on engineering and basic-science research [21]. The overall pathway begins with a clinical need, continues through the development of an engineering solution, and returns to the clinical setting for validation. However, this dependence also creates potential risks. If clinical problems are not defined accurately, or if engineers do not fully understand real operating-room constraints, technological development may become disconnected from clinical needs [16, 22]. Medical imaging has a distinctive bridging role because it is both a core diagnostic tool and the technical basis of robotic navigation [23]. This dual function places imaging journals between the engineering and clinical clusters. The field also shows considerable platform dependence. Research groups often work around specific systems, such as da Vinci, Mona Lisa, Ion, and Monarch. As a result, some findings may reflect the performance of a particular platform rather than general principles of robot-assisted biopsy. This dependence may limit external validity and contribute to the heterogeneity of diagnostic outcomes in the absence of direct head-to-head platform comparisons [5, 16].

### From technical feasibility to AI-driven end-to-end optimization

Prostate cancer is the largest clinical research area within robot-assisted biopsy, reflecting a clear unmet clinical need. Conventional systematic biopsy has long been limited by inadequate detection of clinically significant prostate cancer, overdiagnosis of indolent disease, and infectious complications [24]. Robot-assisted MRI–ultrasound fusion transperineal biopsy systems combine AI-based prostate segmentation with automated needle-path planning and can achieve millimeter-level targeting accuracy. Systems such as Biobot Mona Lisa 2.0 have incorporated AI-based segmentation, automated trajectory planning, and MRI–ultrasound fusion guidance for targeted transperineal biopsy [25].

AI is also extending beyond image guidance into pathological diagnosis. A prospective multicenter study published in npj Digital Medicine demonstrated the clinical value of AI-assisted prostate biopsy reporting in improving diagnostic accuracy, reducing reporting time, and limiting unnecessary testing [26]. The study included 1,613 prostate biopsy cases from three specialist National Health Service centers in the United Kingdom. Staged AI-assisted second review changed the initial diagnosis or grade group in 5.4% of patients, including changes that could affect clinical management in 1.3%. Concurrent AI-assisted review shortened the mean reporting turnaround time by 30.1 h and reduced the need for immunohistochemical testing. These findings indicate that AI assistance is expanding from image interpretation to the entire diagnostic pathway. However, additional systematic cores remain important because approximately one-third of positive cores may be located in the lobe opposite the target lesion [19]. Although robotic fusion systems have been widely recognized for their advantages in improving the detection rate of clinically significant prostate cancer (Grade Group ≥ 2), long-term prospective trials are still needed to firmly establish their superiority over conventional cognitive or visual fusion methods in terms of cost-effectiveness and standardized complication reduction.

### Clinical value in high-risk settings

The importance of robot-assisted stereotactic biopsy is closely related to its role in high-risk anatomical regions where conventional approaches are difficult to perform safely. This clinical role also explains its distinct position in the co-citation network. A systematic analysis of 92 studies included 9,801 frame-based biopsies, 2,665 robot-assisted biopsies, and 1,862 frameless navigation-guided biopsies. The pooled diagnostic yield of robot-assisted biopsy was 97%, compared with 95% for frame-based biopsy and 94% for frameless navigation. The mean procedure time was 76.6 min in the robot-assisted group and was shorter than that of the other approaches. No significant differences were found in symptomatic hemorrhage, temporary or permanent neurological deficits, or mortality [7]. The value of robotic assistance is particularly important in children with brainstem lesions. A systematic review of 15 studies involving 215 patients reported a technical success rate of 99% (95% CI, 98%–100%) and a definitive diagnostic yield of 98%. Temporary postoperative complications occurred in 6% of patients, permanent complications occurred in 1%, and no procedure-related deaths were reported. Subgroup analysis showed similar diagnostic yields for frame-based and frameless systems, supporting robot-assisted stereotactic biopsy as a reliable minimally invasive option for this high-risk population [27]. Recent technological developments include a hybrid pneumatic–hydraulic stereotactic robotic system that combines a large range of motion with high procedural accuracy. The platform may support biopsy, resection, injection, ablation, stereoelectroencephalography, and deep-brain stimulation electrode placement [28, 29].

### The fastest-growing emerging frontier

Thoracic intervention is one of the most rapidly expanding areas of robot-assisted biopsy and represents the extension of the technology into new anatomical settings. The increasing use of low-dose CT screening has led to the detection of more small and peripherally located pulmonary nodules. Conventional bronchoscopy may not reliably reach these distal lesions, while transthoracic biopsy has a higher risk of pneumothorax and bleeding. Shape-sensing robot-assisted bronchoscopy provides a minimally invasive alternative by improving catheter flexibility, stability, and access to peripheral lesions. Its overall diagnostic yield is higher than that of virtual bronchoscopic navigation, while procedure-related complications are less frequent [30].

When shape-sensing robot-assisted bronchoscopy is combined with cone-beam CT and formalin-fixed, paraffin-embedded histopathological assessment, the diagnostic yield can exceed 90%. This highlights the importance of real-time intraoperative imaging and strict pathological criteria. For peripheral pulmonary nodules with a median diameter of only 13 mm, the combination of robotic bronchoscopy and cone-beam CT achieved a strict diagnostic yield of 85% and a diagnostic accuracy of 92.0% [31]. Nodule appearance and location on CT did not significantly affect diagnostic yield, suggesting that a stable robotic catheter platform may broaden the range of accessible lesions. The clinical introduction of robot-assisted bronchoscopy has also been associated with increased detection of early-stage lung cancer, supporting its potential role in the diagnostic pathway following lung cancer screening.

In transthoracic biopsy, the first-in-human study of a patient-mounted robotic needle-guidance platform reported technical and clinical success rates above 93%. Adequate tissue was obtained in all cases for histopathological examination and molecular profiling, and no major complications occurred [32]. With increasing operator experience, the median procedure time decreased from 61.4 to 30.5 min, together with a reduction in radiation exposure. Similar improvements were observed among operators with different levels of experience, suggesting that robotic platforms may shorten the learning curve and reduce operator dependence.

### From image guidance to intelligent support across the clinical pathway

Image-guided systems have traditionally provided anatomical information to clinicians. The field is now entering an era of AI-guided intervention. AI-guided needle-placement robots can be organized into four interconnected modules: anatomical perception, motion planning, instrument-state sensing, and motion execution [33]. AI can analyze medical images, identify targets and obstacles, calculate safe trajectories, and guide a needle toward the target with high precision. Phantom experiments have shown that AI-based lesion-detection and path-planning systems can identify target lesions and generate feasible needle trajectories, with better targeting accuracy and procedural efficiency than manual puncture [34]. However, the first-in-human experience also indicated that the cost function used for automated trajectory ranking requires further optimization before routine integration into clinical workflows [32, 35].

Under current autonomy frameworks, most robotic systems operate at the task-autonomy level. They can independently perform predefined subtasks, but clinicians retain control over key decisions. Progress toward higher autonomy will require real-time compensation for tissue deformation, integration of multimodal sensing data, and clinical validation of explainable AI algorithms [9]. Full autonomy may not be the only or most suitable pathway. Low-complexity robotic devices that preserve operator control over needle insertion may offer advantages in clinical acceptability and safety [36]. A fully autonomous robotic ultrasound-guided biopsy framework has undergone preliminary validation for superficial organs, but further clinical evaluation is still required [37].

### From proof of concept to preclinical investigation

Although micro- and nanorobots show considerable potential for targeted drug delivery and minimally invasive diagnosis, the bibliometric findings indicate that this research remains largely exploratory. Several recent preclinical advances suggest that the field is beginning to move from proof-of-concept experiments toward animal studies and investigations using human specimens. In neurosurgery, a three-dimensional neuronavigated microrobot platform enabled the deployment of a flexible biopsy needle in deep brain regions. Preclinical studies successfully obtained tissue samples from cadaveric and sheep models, and pathological assessment showed less tissue injury along the insertion tract than with conventional stereotactic needles [38].

In pulmonary intervention, a magnetic microrobot with combined drilling and sensing functions demonstrated the feasibility of targeted biopsy of deep tracheal microlesions in ex vivo porcine lungs and in vivo rabbit models. The Helixoft helical magnetic soft microrobot reported in Nature Communications achieved controlled active steering within a 300-μm catheter using magnetic actuation [39]. At the cellular level, an automated nanoprobe platform enabled label-free extraction of mitochondria from living cells, extending robot-assisted sampling from tissues to intracellular organelles [40]. Although these advances involve different anatomical targets and technical approaches, they point toward a common direction. Micro- and nanorobots are progressing from controlled movement in laboratory environments toward the performance of meaningful biomedical tasks under physiological conditions.

## Limitations

This study has several limitations. First, the primary analysis was based on English-language publications indexed in the Web of Science Core Collection. Studies from other databases, non-English publications, and grey literature may therefore have been missed. Second, the terminology surrounding robot-assisted, computer-assisted, navigation-guided, and stereotactic biopsy overlaps considerably. Although the data were standardized, some borderline studies may have been misclassified. Third, older publications have had more time to accumulate citations, whereas the academic influence of recent studies may have been underestimated. Variations in author and institution names may also have affected the collaboration networks. Finally, bibliometric indicators reflect academic attention and knowledge dissemination rather than actual diagnostic effectiveness. The independent PubMed MeSH analysis provided additional validation from a biomedical semantic perspective, but it could not fully eliminate these limitations.

## Conclusion

Robot-assisted biopsy is evolving from a technically feasible procedure into an integrated diagnostic platform supported by image fusion, clinical decision-making, and intelligent human–robot collaboration. Prostate biopsy represents the most mature pathway of clinical translation, while robot-assisted bronchoscopy is the most prominent emerging direction. Human–robot collaboration supported by imaging and multimodal sensing may define the next stage of technological development.The central question is no longer whether a robot can place a biopsy needle more accurately, but whether this accuracy can produce more adequate specimens, reduce repeat procedures, lower patient risk, and support better treatment decisions. Multicenter comparative studies, standardized outcome measures, transparent validation of AI systems, and health-economic evaluations will be essential for integrating robot-assisted biopsy into routine clinical practice.

## Supplementary Information

Below is the link to the electronic supplementary material.Supplementary file 1

## Data Availability

No datasets were generated or analysed during the current study.
