# Supplementary material for: Robot-assisted biopsy a bibliometric analysis of global trends, hotspots, and emerging frontiers (2000–2025)
Source: J Robot Surg. 2026 Aug 28;20(1):901. doi: 10.1007/s11701-026-03889-2 (PMC13525022; doi:10.1007/s11701-026-03889-2)
Supplement: Supplementary file 1 [file 11701_2026_3889_MOESM1_ESM.docx]

**1. Detailed search strategies**

**Web of Science Core Collection (WoSCC; Science Citation Index Expanded)**

TS=((robot* OR "robot-assisted" OR "robot assisted" OR "robotic-assisted" OR "robotic assisted" OR "robotic system*" OR "robotic platform*" OR "robotic guidance" OR "robotic-guided" OR "robotic navigation") AND (biopsy OR "needle biopsy" OR "core needle biopsy" OR "tissue sampling" OR "needle insertion" OR "percutaneous biopsy" OR "image-guided biopsy" OR "image guided biopsy"))

**Restrictions:** publication date from January 1, 2000 to December 31, 2025; English language; document types Article and Review. Records were exported as Full Record and Cited References in Web of Science plain-text format.

**Final search and export date:** May 25, 2026.

**Record flow:** The initial search returned 2,779 records. After the English-language and Article/Review filters, 2,520 records remained. Subject-category and topic-relevance screening produced the final WoSCC export of 2,336 records. The supplied export already met the prespecified year and document-type criteria; the subsequent structural audit removed no additional records or duplicates.

**PubMed**

((("Robotic Surgical Procedures"[MeSH] OR "Robotics"[MeSH] OR robot*[Title/Abstract] OR "robot-assisted"[Title/Abstract] OR "robot assisted"[Title/Abstract] OR "robotic-assisted"[Title/Abstract] OR "robotic assisted"[Title/Abstract] OR "robotic system"[Title/Abstract] OR "robotic systems"[Title/Abstract] OR "robotic platform"[Title/Abstract] OR "robotic platforms"[Title/Abstract]) AND ("Biopsy"[MeSH] OR "Image-Guided Biopsy"[MeSH] OR biopsy[Title/Abstract] OR "needle biopsy"[Title/Abstract] OR "core needle biopsy"[Title/Abstract] OR "percutaneous biopsy"[Title/Abstract] OR "image-guided biopsy"[Title/Abstract] OR "image guided biopsy"[Title/Abstract])) AND english[Language] AND 2000:2025[Date - Publication])

**Restrictions:** publication date from January 1, 2000 to December 31, 2025; English language; publication type Journal Article or Review. PubMed records were retained in native MEDLINE/PubMed tagged-text format.

**Final search and export date:** May 25, 2026.

**Record flow:** The source export contained 1,592 records. Thirty records with an ineligible publication type and 30 records outside the final 2000-2025 window or without an eligible year were excluded. One duplicate publication was removed by DOI/normalized-title matching. The final independent PubMed dataset comprised 1,531 records, all with a unique PMID and at least one MeSH heading.

**2. Study selection and deduplication procedure**

**Eligibility criteria**

**Inclusion criteria were:** (1) publication from 2000 through 2025; (2) English-language record; (3) an Article or Review in WoSCC, or a Journal Article or Review in PubMed; and (4) direct relevance to the design, validation, navigation, control, or clinical application of robotic systems for tissue biopsy, needle sampling, bronchoscopic sampling, or stereotactic biopsy.

**Exclusion criteria were:** (1) conference papers, proceedings papers, book chapters, editorials, letters, corrections, news items, meeting abstracts, retracted publications, and preprint-only records; (2) records outside 2000-2025; (3) records unrelated to robot-assisted tissue sampling after title/abstract or full-record assessment; (4) studies limited to robot-assisted therapy without biopsy, liquid biopsy, automated laboratory assays, or non-robotic conventional puncture; and (5) records lacking the identifier or bibliographic information required for the planned analysis.

Two investigators screened records independently using the prespecified criteria. Full bibliographic records were reviewed when title and abstract information was insufficient, and disagreements were resolved by discussion and consensus.

**Deduplication and database separation**

Duplicates were checked within each database only. WoSCC records were matched primarily by the unique WoS accession number (UT), with DOI and normalized title as secondary fields. PubMed records were matched first by PMID and then by DOI and normalized title. WoSCC and PubMed were not merged, and no cross-database deduplication was performed. WoSCC was used for the primary bibliometric analyses; PubMed was used independently for supplementary MeSH descriptor and qualifier analyses.

| **Database processing step** | **Records** |
| --- | --- |
| WoSCC initial search | 2,779 |
| WoSCC after language and document-type filters | 2,520 |
| WoSCC final screened and cleaned dataset | 2,336 |
| WoSCC duplicates removed during final structural audit | 0 |
| PubMed source export | 1,592 |
| PubMed excluded: publication type | 30 |
| PubMed excluded: year outside 2000-2025 or ineligible/missing year | 30 |
| PubMed duplicate publication removed | 1 |
| PubMed final independent MeSH dataset | 1,531 |

**3. Data cleaning and validation**

**WoSCC bibliographic fields**

The final WoSCC export contained 2,336 unique UT records spanning 2000-2025. No record had a missing UT, publication year, document type, abbreviated-author field, or full-author field. Institutional addresses were missing for three records and cited references for two records; these publications were retained because missing non-key fields were not exclusion criteria. A total of 89,195 cited-reference lines from 2,334 records were preserved for co-citation and dual-map analyses.

Author names were reconciled using AU, AF, C1, UT, and, where available, ORCID or correspondence evidence. AU was normalized to SURNAME, INITIALS and AF to SURNAME, FULL GIVEN NAME. Unambiguous split fragments were merged; collective names and unverifiable incomplete fragments were removed from author-network fields while the publications themselves were retained. The final AU and AF fields each contained 17,116 entries.

Institutional strings were standardized to the principal university, hospital, or research-organization level. Corresponding-author prefixes, personal-name fragments, and generic department-only labels were removed. Country variants were mapped to canonical names, and journal titles were harmonized for capitalization and abbreviations.

Author keywords (DE) and Keywords Plus (ID) were cleaned with an auditable thesaurus. Capitalization, punctuation, hyphenation, singular/plural forms, spelling variants, abbreviations, and confirmed synonyms were normalized. Generic or non-informative terms were removed. Cleaning actions comprised 1,977 synonym mappings, 2,842 format normalizations, and 2,702 noise-term removals; 17,356 terms were unchanged. After cleaning, 11,321 DE terms and 10,718 ID terms remained. The 552 records flagged for manual topic review were retained; keyword cleaning was not used as a substitute for article-level relevance screening.

**PubMed MeSH fields**

The native PubMed records contained 18,768 MeSH-heading fields representing 1,598 unique descriptors. Author-keyword fields (OT) were excluded from the MeSH-only file so that the analysis used controlled vocabulary only. Official MeSH descriptors and qualifiers were preserved exactly and were not stemmed or synonym-merged.

For the final VOSviewer MeSH file and thematic figures, 50 generic descriptors covering demographic, species, geography, broad study-design, statistical, and other non-topic-specific concepts were excluded. This removed 6,799 MeSH lines and retained 11,969 MeSH fields; all 1,531 records retained at least one MeSH heading. Of the 1,598 valid descriptors, 333 were assigned to six prespecified semantic domains for the focused figures, while 1,215 valid descriptors were retained in the cleaned dataset but were not used in the six-domain visualizations.

| **Validation item** | **Result** |
| --- | --- |
| WoSCC final records / unique UT | 2,336 / 2,336 |
| WoSCC cited-reference coverage | 2,334 records; 89,195 CR lines |
| WoSCC final AU / AF entries | 17,116 / 17,116 |
| PubMed final records / unique PMID | 1,531 / 1,531 |
| PubMed records with at least one MeSH heading | 1,531 (100%) |
| PubMed raw MeSH fields / unique descriptors | 18,768 / 1,598 |
| Generic MeSH descriptors excluded | 50 descriptors; 6,799 lines |
| MeSH fields retained after focused cleaning | 11,969 |

**4. Software, parameters, and compatible output formats**

**VOSviewer settings**

| **Parameter** | **Setting** |
| --- | --- |
| Software version | VOSviewer 1.6.20 |
| Counting method | Full counting |
| Normalization method | Association strength |
| Country co-authorship threshold | Minimum 2 publications |
| Institution co-authorship threshold | Minimum 5 publications |
| Author co-authorship threshold | Minimum 5 publications |
| Keyword/MeSH co-occurrence threshold | Minimum 10 occurrences |
| Journal co-citation threshold | Minimum 50 citations |
| Cited-reference co-citation threshold | Minimum 20 citations |
| Clustering | VOS clustering; resolution 1.0; minimum cluster size 2 |

The cleaned WoSCC file retained native Web of Science plain-text tags, three-space continuation lines for repeated AU/AF/C1/CR values, one ER marker per record, and one terminal EF marker. It can be imported directly into VOSviewer as a Web of Science bibliographic database file and into CiteSpace as Web of Science plain text.

The cleaned PubMed MeSH file retained native PubMed/MEDLINE tags and contained no Web of Science wrapper markers. It can be imported directly into VOSviewer as a PubMed bibliographic database file. The MeSH-only file excludes OT author keywords to prevent controlled descriptors and free-text keywords from being counted together.

**CiteSpace settings**

| **Parameter** | **Setting** |
| --- | --- |
| Software version | CiteSpace 6.4.R1 |
| Time span | 2000-2025 |
| Time slicing | 1 year per slice |
| Primary analyses | Cited-reference burst detection; journal dual-map overlay |
| Selection criteria | g-index (k = 25) |
| LRF | 2.5 |
| L/N | 10 |
| LBY | 5 |
| e | 1 |
| Burst detection gamma | 1 |
| Minimum burst duration | 2 years |
| Pruning | Pathfinder and pruning sliced networks, where applicable |
